# Elemental Composition, Phosphorous Uptake, and Characteristics of Growth of a SAR11 Strain in Batch and Continuous Culture

**DOI:** 10.1128/mSystems.00218-18

**Published:** 2019-05-21

**Authors:** Scott R. Grant, Matthew J. Church, Sara Ferrón, Edward A. Laws, Michael S. Rappé

**Affiliations:** a Department of Oceanography, School of Ocean and Earth Science and Technology, University of Hawaii at Manoa, Honolulu, Hawai’i, USA; b Flathead Lake Biological Station, University of Montana, Polson, Montana, USA; c Daniel K. Inouye Center for Microbial Oceanography: Research and Education, School of Ocean and Earth Science and Technology, University of Hawaii at Manoa, Honolulu, Hawai’i, USA; d Department of Environmental Sciences, College of the Coast and Environment, Louisiana State University, Baton Rouge, Louisiana, USA; e Hawaii Institute of Marine Biology, School of Ocean and Earth Science and Technology, University of Hawaii at Manoa, Kaneohe, Hawai’i, USA; Purdue University

**Keywords:** SAR11, bacterial production, biogeochemistry, marine microbiology, phosphorous, respiration, seawater

## Abstract

In this study, a strain of SAR11 subgroup IIIa (termed HIMB114) was grown in seawater-based batch and continuous culture in order to quantify cellular features and metabolism relevant to SAR11 ecology. We report some of the first direct measurements of cellular elemental quotas for nitrogen (N) and phosphorus (P) for SAR11, grown in batch culture: 1.4 ± 0.9 fg N and 0.44 ± 0.01 fg P, respectively, that were consistent with the small size of HIMB114 cells (average volume of 0.09 μm^3^). However, the mean carbon (C) cellular quota of 50 ± 47 fg C was anomalously high, but variable. The rates of phosphate (PO_4_^3−^) uptake measured from both batch and continuous cultures were exceptionally slow: in chemostats growing at 0.3 day^−1^, HIMB114 took up 1.1 ± 0.3 amol P cell^−1^ day^−1^, suggesting that <30% of the cellular P requirement of HIMB114 was met by PO_4_^3−^ assimilation. The mean rate of leucine incorporation, a measure of bacterial production, during late-log-phase growth of batch HIMB114 cultures was 0.042 ± 0.02 amol Leu cell^−1^ h^−1^. While only weakly correlated with changes in specific growth rates, the onset of stationary phase resulted in decreases in cell-specific leucine incorporation that were proportional to changes in growth rate. The rates of cellular production, respiratory oxygen consumption, and changes in total organic C concentrations constrained cellular growth efficiencies to 13% ± 4%. Hence, despite a small genome and diminutively sized cells, SAR11 strain HIMB114 appears to grow at efficiencies similar to those of naturally occurring bacterioplankton communities.

**IMPORTANCE** While SAR11 bacteria contribute a significant fraction to the total picoplankton biomass in the ocean and likely are major players in organic C and nutrient cycling, the cellular characteristics and metabolic features of most lineages have either only been hypothesized from genomes or otherwise not measured in controlled laboratory experimentation. The dearth of data on even the most basic characteristics for what is arguably the most abundant heterotroph in seawater has limited the specific consideration of SAR11 in ocean ecosystem modeling efforts. In this study, we provide measures of cellular P, N, and C, aerobic respiration, and bacterial production for a SAR11 strain growing in natural seawater medium that can be used to directly relate these features of SAR11 to biogeochemical cycling in the oceans. Through the development of a chemostat system to measure nutrient uptake during steady-state growth, we have also documented inorganic P uptake rates that allude to the importance of organic phosphorous to meet cellular P demands, even in the presence of nonlimiting PO_4_^3−^ concentrations.

## Abstract (precis)

While SAR11 bacteria contribute a significant fraction to the total picoplankton biomass in the ocean and likely are major players in organic C and nutrient cycling, the cellular characteristics and metabolic features of most lineages have either only been hypothesized from genomes or otherwise not measured in controlled laboratory experimentation. The dearth of data on even the most basic characteristics for what is arguably the most abundant heterotroph in seawater has limited the specific consideration of SAR11 in ocean ecosystem modeling efforts. In this study, we provide measures of cellular P, N, and C, aerobic respiration, and bacterial production for a SAR11 strain growing in natural seawater medium that can be used to directly relate these features of SAR11 to biogeochemical cycling in the oceans. Through the development of a chemostat system to measure nutrient uptake during steady-state growth, we have also documented inorganic P uptake rates that allude to the importance of organic phosphorous to meet cellular P demands, even in the presence of nonlimiting PO_4_^3−^ concentrations.

## INTRODUCTION

The SAR11 bacterial lineage is a genetically diverse clade of aquatic, free-living cells with compact, streamlined genomes, found broadly distributed throughout the oceans (1). They are also among the smallest free-living cells from the ocean for which there are isolated strains available to study in the laboratory (2). Typical biovolumes for healthy SAR11 cells range from 0.015 to 0.058 μm^3^ (3), and the cells possess a crescent shape (2–5). Small cells are thought to have an advantage in oligotrophic environments, where they should be able to outcompete larger osmotrophs for nutrients relative to their requirements for growth, ascribed to the importance of having a large surface area-to-volume ratio (6).

Culture studies examining the physiology of SAR11 strains have provided a number of unexpected discoveries and valuable insights into the metabolism of the clade (1). Directed by clues generated from genome analysis indicating that a number of metabolic pathways common to chemoheterotrophs were incomplete or missing, subsequent culture studies led to evidence of unusual growth requirements for SAR11 (5, 7–12). For example, evidence of an incomplete assimilatory sulfate reduction pathway led Tripp and colleagues to the discovery that SAR11 strain HTCC1062 requires a source of reduced sulfur for growth, which could be satisfied by methionine or dimethylsulfoniopropionate (7). Further investigations showed that SAR11 had a variant of the standard glycolysis pathways, with nonconserved ability of SAR11 strains to oxidize simple sugars, while low-molecular-weight organic acids were shown to be important carbon sources for many SAR11 strains (9). In subsequent experiments, Carini and colleagues were able to successfully grow SAR11 strain HTCC1062 on a novel defined artificial seawater medium with pyruvate serving as a C source, methionine as the sole sulfur source, and glycine as a necessary amino acid, along with standard base salts, inorganic macronutrients PO_4_^3−^ and ammonium (NH_4_^+^), and micronutrient trace metal and vitamin additions (5). Laboratory experiments with isolated SAR11 strains have primarily focused on representatives from the SAR11 subclade Ia, which includes the majority of isolates, including “*Candidatus* Pelagibacter ubique” strain HTCC1062 (2), with little information from representatives of other SAR11 subclades.

Recent studies suggest that the type of phosphorus available, whether present as PO_4_^3−^ or dissolved organic P (DOP), is an important control on microbial niche partitioning in the sea (13, 14). The Global Ocean Sampling (GOS) expedition, an extensive metagenomic survey of marine surface waters, revealed that genes from the high-affinity PO_4_^3−^ transport system (*pstS*) most closely matching sequenced *Prochlorococcus* and SAR11 genes, were among the most highly recruited annotated genes (15). Moreover, during the GOS expedition, *pstS* genes were the single most significant difference between the tropical Atlantic and equatorial Pacific samples, differing by a factor of more than seven in relative abundance (15). Studies of culture representatives of *Prochlorococcus*, the most abundant oxygenic photoautotroph in the ocean, confirm that there appear to be substantial differences in the presence, topology, and regulation of genes thought to be involved in P acquisition between strains of *Prochlorococcus* (16), with different strains able to metabolize inorganic versus labile organic P compounds. Finally, in a gene content comparison of whole-population genomes of *Prochlorococcus* and SAR11 between microbial communities inhabiting the well-known stations of the Hawaii Ocean Time-series (HOT) program (North Pacific) and the Bermuda Atlantic Time-series Study (BATS) (North Atlantic), Coleman and Chisholm found that of the 1.8% of gene clusters that had significant abundance differences between the Atlantic and Pacific populations, 87% of those genes were involved in PO_4_^3−^ or phosphonate metabolism (17).

Motivated by the intriguing evidence that P acquisition strategies are under strong selection pressure and may be a potential dimension over which SAR11 lineages are differentiated, this study sought to investigate the uptake capability of the SAR11 subclade IIIa isolate HIMB114 for PO_4_^3−^. Because SAR11 bacteria characteristically dominate marine planktonic microbial communities, it is also a notable deficiency that typical parameters needed to model their growth and response under variable environmental conditions are not yet available. Thus, this study also sought to measure a number of basic cellular properties, such as elemental composition, and physiological rate measurements, including cellular production, respiration, and growth efficiency. In the process, a continuous culture of an axenic SAR11 strain was developed for the first time, enabling assessment of many of these physiological features under defined growth conditions.

## RESULTS

### Culture growth and cell size.

**FIG 1:**
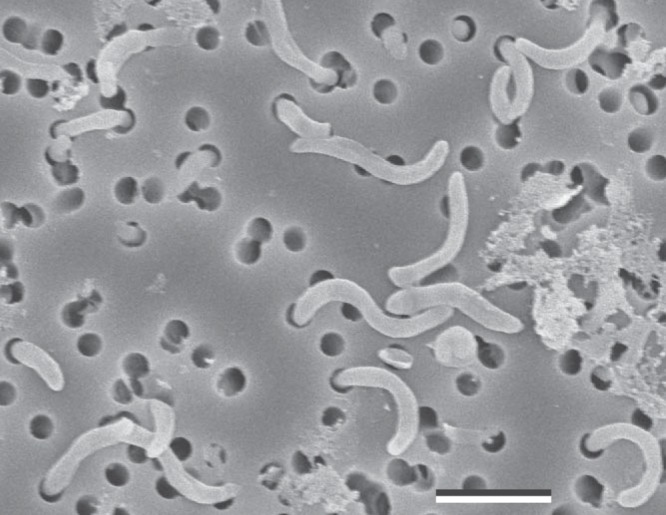
Scanning electron micrograph of HIMB114 cells growing in early stationary-phase batch culture. Bar, 1 μm.

In natural seawater-based growth medium, strain HIMB114 reached a maximum specific growth rate of 1.2 day^−1^ and yielded 5 × 10^5^ to 8 × 10^5^ cells ml^−1^ (see Fig. S1 in the supplemental material). HIMB114 cells were observed to have a crescent shape that was consistent with previous microscopic observations of SAR11 (Fig. 1). Elongated cells of strain HIMB114 were observed that consisted of spirillum morphologies of two to four “regular” (i.e., recently divided) single-cell lengths. These longer cell morphologies were a small fraction (few percent) of the cells during exponential growth phase but became an increasing percentage (up to 30%) of cells as the culture entered into stationary phase. Distributions of cell size parameters for length, width, and biovolume were all nonnormal and fit as log-normal distributions to calculate the most frequent and mean size parameter values (Fig. S2). The mean of the distribution was used when normalizing any quantities to a cell size parameter (length, 1.07 μm; width, 0.32 μm; volume, 0.09 μm^3^).

10.1128/mSystems.00218-18.3 FIG S1 Batch culture growth curves for six replicate 10-liter cultures of strain HIMB114. Lower error bars represent the standard deviations among count fields (typically 10 to 15), while upper error bars represent the cell density if morphologies representing multiple cells are converted to single cell units. The mean regression slope for exponential specific growth rate for the cultures is 1.08 (SD, 0.03) day^−1^. Strains were grown in standard medium at 26**°**C. Download FIG S1, PDF file, 0.1 MB. Copyright © 2019 Grant et al. 2019 Grant et al. This content is distributed under the terms of the Creative Commons Attribution 4.0 International license.

10.1128/mSystems.00218-18.4 FIG S2 Cell size analysis of SAR11 strain HIMB114. Distribution of length (top), width (middle), and biovolume (bottom) for 769 cells, with fit to log-normal distribution. Download FIG S2, PDF file, 0.2 MB. Copyright © 2019 Grant et al. 2019 Grant et al. This content is distributed under the terms of the Creative Commons Attribution 4.0 International license.

### Cellular elemental quotas.

The P cell quota for strain HIMB114 measured in batch cultures from early stationary phase was 14.2 ± 0.4 amol P cell^−1^ (mean ± standard deviation [SD]; *n* = 6) or 0.44 fg P cell^−1^, with a mean precision of 2% for triplicate 4- to 5-liter culture volumes. The particulate P controls made from spent media were 5% of that measured for the cellular biomass collected on their corresponding 0.2-μm-pore-size membrane filter. Hence, the modified method for measuring particulate P on 47-mm-diameter PC membranes described in Materials and Methods appeared to work well.

Filtered medium blanks for particulate C and N were high relative to the sample signal and increased with the volume of medium filtered (Fig. S3). Because complete saturation was not conclusive even at a medium blank volume of 10 liters, rectangular hyperbolic saturation functions were fit by nonlinear least-squares regression to the blank C and N data versus filtered medium volume in order to extrapolate the associated blank values for the 30 liters of total volume filtered (Fig. S3). After normalizing to the total number of cells captured on each filter, the mean C cell quota was 50 ± 47 fg C cell^−1^ (mean ± SD; *n* = 3) or 4.2 fmol C cell^−1^, and the mean N cell quota was 1.4 ± 0.9 fg N cell^−1^ (mean ± SD; *n* = 3) or 0.1 fmol N cell^−1^.

10.1128/mSystems.00218-18.5FIG S3Filtered medium blanks for particulate carbon and nitrogen measurements as a function of volume of medium filtered. Filtered medium (47-mm-diameter, 0.2-μm-pore-size Nuclepore PC membrane followed by 0.22-μm-pore-size, Sterivex-GP polyethersulfone membrane) was used as the source for procedural blanks filtered through combusted GF-75 glass fiber filters to correct for adsorption of dissolved organic carbon (top) or inorganic nitrogen (bottom). Carbon data were fit to a rectangular hyperbolic saturation function: Blank = $8.5(\text{μg C})+\frac{43.5(\text{μg C})\cdot V}{1.9(L)+V}$ for medium volume (*V*) (in liters). Nitrogen data were fit to a rectangular hyperbolic saturation function: Blank = $1.1(\text{μg N})+\frac{8.1(\text{μg N})\cdot V}{2.4(L)+V}$ for medium volume (*V*) (in liters). Download FIG S3, PDF file, 0.1 MB.Copyright © 2019 Grant et al.2019Grant et al.This content is distributed under the terms of the Creative Commons Attribution 4.0 International license.

### PO_4_^3−^ uptake in batch and continuous culture.

**FIG 2:**
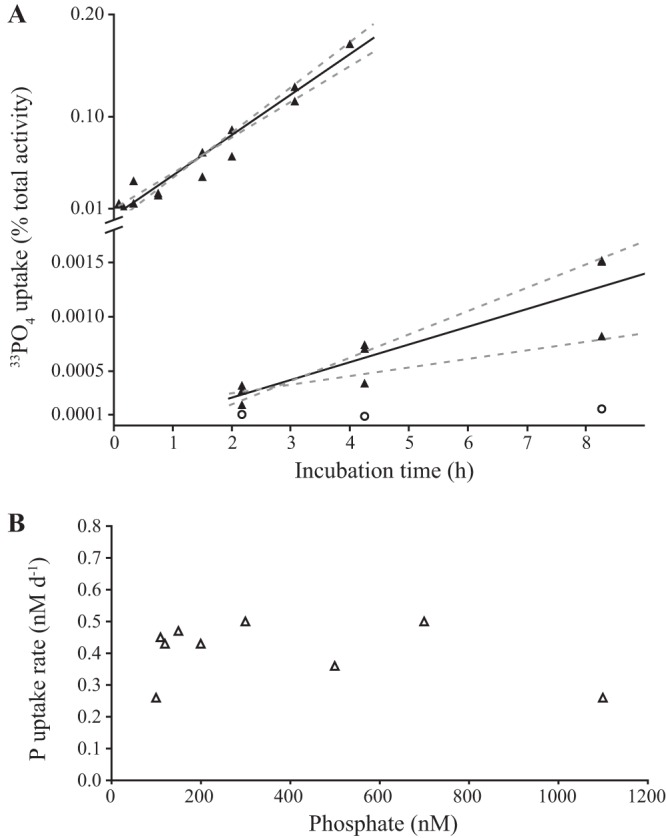
Rates of PO_4_^3−^ uptake by SAR11 strain HIMB114. (A) Time course measurements of [^33^P]phosphate uptake in a chemostat culture of strain HIMB114 (top left line) as well as a batch culture (bottom right line) with blank controls (circles). Solid lines indicate the linear least-squares regression, while dashed lines indicate the 95% prediction confidence bands. (B) [^33^P]phosphate uptake kinetics for a chemostat culture of HIMB114, calculated from single time point, 22-h incubations across a range of phosphate concentrations.

The rates of PO_4_^3−^ uptake measured by a ^33^P radiotracer for a continuous culture of strain HIMB114 were extremely low (Fig. 2A). Of the five PO_4_^3−^ uptake rate time course measurements performed from the chemostat cultures over 4 to 6 h at ambient (100 nmol liter^−1^) phosphate concentrations, the mean specific uptake rate was 0.007 ± 0.0025 day^−1^ (mean ± SD; *n* = 5), with a mean coefficient of determination of uptake versus time of 0.97 (Fig. 2A). This corresponds to a mean PO_4_^3−^ turnover time (*T_P_*) of 160 ± 50 days (mean ± SD; *n* = 5), or a bulk PO_4_^3−^ uptake rate of 0.68 nmol liter^−1^ P day^−1^. In cell-specific units, strain HIMB114 took up 1.1 ± 0.3 amol P cell^−1^ day^−1^ (mean ± SD; *n* = 5), or less than 10% of the cellular P quota per day, despite growing at a rate of 0.3 day^−1^. Phosphate uptake kinetics measured for the chemostat culture averaged 0.4 ± 0.09 nmol liter^−1^ P day^−1^ (mean ± SD; *n* = 9) across all PO_4_^3−^ additions, showing no significant correlation between uptake rate and PO_4_^3−^ concentration within the measurement error (Fig. 2B). This observation most likely reflects the fact that the culture was not P limited, an interpretation confirmed by the fact that PO_4_^3−^ additions to batch cultures entering stationary phase had no effect on growth (not shown).

For the HIMB114 strain grown under batch conditions, PO_4_^3−^ uptake rates were also extremely low (Fig. 2A). Measured during late exponential phase for a culture growing at 1.02 day^−1^, the highest specific uptake rate measured was 4 × 10^−5^ day^−1^, equivalent to a turnover time of the PO_4_^3−^ pool of 70 years (ranging from 50 to 100 years). In bulk units, the maximum PO_4_^3−^ uptake rate for the batch cultures in late exponential growth was 6 pmol P liter^−1^ day^−1^. To confirm that the cells were actively growing, leucine incorporation measurements were conducted at the same time as the PO_4_^3−^ uptake measurements (described in greater detail below). The resulting production rate was 37 ± 2.6 nmol C liter^−1^ day^−1^ (mean ± SD; *n* = 4) that, when converted to P units using a 75:1 C:P molar ratio, yields a P requirement of 0.5 nmol P liter^−1^ day^−1^. Given that the measured bulk PO_4_^3−^ uptake rate was 6 pmol P liter^−1^ day^−1^ (or ∼1% of the requirement), such results suggest that PO_4_^3−^ was not the primary source of P for HIMB114 growing on natural seawater-based medium containing 100 to 150 nmol PO_4_ liter^−1^.

### Chemostat steady-state theory.

The theoretical expectations for PO_4_^3−^ uptake rate and turnover time measurements for the chemostat system are fairly well constrained, much better than for batch culture growth, because steady-state theory may be applied (18). The cell-specific uptake rate is the product of the specific growth rate (μ) with the cellular P quota (*Q_P_*): *V* = μ*Q_P_*. The growth rate is experimentally set by the chemostat dilution rate, here 0.3 day^−1^. The cellular P quota (measured at 14.2 amol P cell^−1^) yielded a theoretical uptake rate of 4.3 ± 0.5 amol P cell^−1^ day^−1^. In comparison, the highest measured uptake rate was 1.3 ± 0.2 amol P cell^−1^ day^−1^, or about 30% of the theoretical value. This was the highest uptake rate measured for the culture and, consistent with results from the batch culture, indicated that the HIMB114 strain growing under steady-state conditions with PO_4_^3−^ concentrations at 100 nmol liter^−1^ was likely not using PO_4_^3−^ as the sole or primary P source for growth and was instead meeting a large fraction of its P requirements from assimilation of organic P.

### Bacterial production.

**FIG 3:**
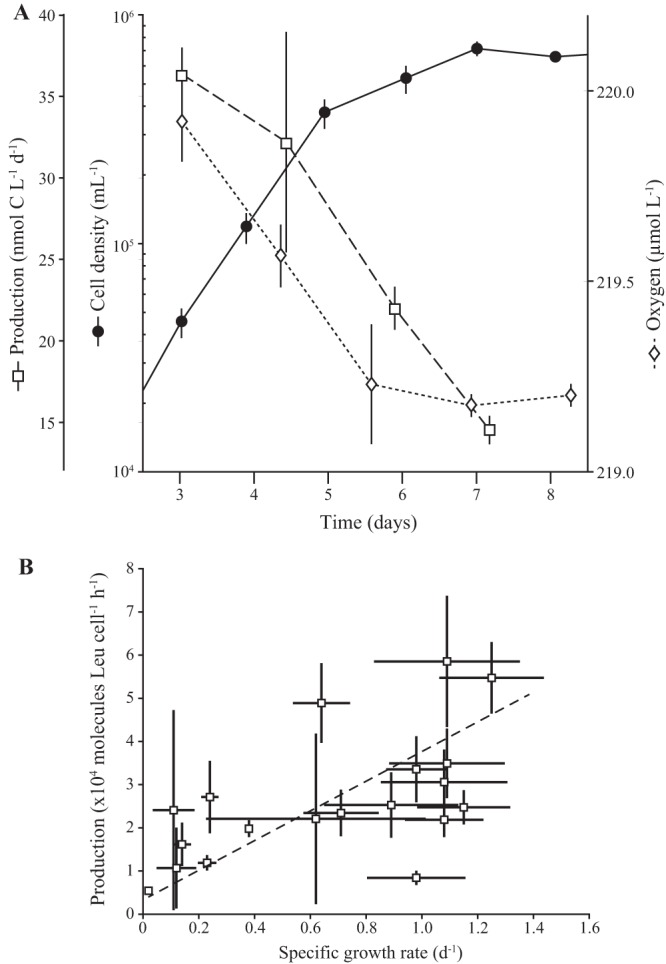
Production and respiration of strain HIMB114 during growth in batch culture. (A) Cellular growth (filled circles), bacterial production ([^3^H]Leu; open squares), and dissolved oxygen concentrations (open diamonds) for a 10-liter batch culture of HIMB114 measured throughout late exponential and into stationary phase (Table 2, experiment 1). (B) Bacterial production, expressed as molecules leucine per cell per hour over 2-h incubations, versus daily specific growth rates for the same batch cultures of strain HIMB114, calculated by changes in cell densities between cultures sampled 1 day apart. The slope of the fit line between production and daily specific growth rate is (0.8 ± 0.3) (95% CI) × 10^6^ molecules Leu cell^−1^.

Bacterial production measurements were conducted on four consecutive days spanning the end of log phase into the early transition to stationary phase from the five batch culture experiments (Fig. 3A and Fig. S4). The mean per cell rate of leucine incorporation across all cultures grown on K-Bay standard medium was 0.042 ± 0.02 amol Leu cell^−1^ h^−1^ (mean ± SD; *n* = 20), resulting in average cell-specific rates of production of 0.13 ± 0.07 fmol C cell^−1^ day^−1^ (mean ± SD; *n* = 20) (Table 1).

**TABLE 1:** Mean, minimum, and maximum bacterial production rates for SAR11 strain HIMB114 grown on sterilized K-Bay seawater medium^*a*^

Bacterial production rate	Mean ± SD	Maximum	Minimum
pmol Leu liter^−1^ h^−1^	16 ± 6.5	28	5
amol Leu cell^−1^ h^−1^	0.04 ± 0.02	0.1	0.01
μg C liter^−1^ day^−1^	0.6 ± 0.2	1.0	0.2
fmol C cell^−1^ day^−1^	0.13 ± 0.07	0.29	0.3

a Production (*n* = 20) was measured in both batch and chemostat cultures by [^3^H]leucine incorporation and converted to C units with a leucine-to-C conversion factor of 1.5 kg C mol Leu^−1^.

10.1128/mSystems.00218-18.6 FIG S4 Bacterial production versus batch culture growth of strain HIMB114. Bacterial production (measured by [^3^H]Leu incorporation) during batch culture growth of a single 10-liter culture of strain HIMB114, sampled throughout late exponential phase and into stationary phase. Open circles and dotted line indicate cell density, while filled squares and solid line indicate bacterial production. Download FIG S4, PDF file, 0.10 MB. Copyright © 2019 Grant et al. 2019 Grant et al. This content is distributed under the terms of the Creative Commons Attribution 4.0 International license.

Bacterial production measurements for strain HIMB114 were relatively uniform, with a coefficient of variation of 40% across the 10 different cultures and 20 independent measurements for cells grown on natural K-Bay seawater medium, despite being measured across a range of growth rates throughout exponential and early stationary phases of batch growth (linear correlation coefficient of 0.58; *P* value of 0.009) (Fig. 3B). However, in individual batch culture experiments, a decline in rate of cell division associated with entry into stationary phase was associated with a concomitant decline in production measured by leucine incorporation (Fig. 3A and Fig. S4), and the slope of the fit line for the plot of bacterial production versus specific growth rate was positive (0.8 ± 0.3 [95% confidence interval {95% CI}]) × 10^6^ molecules Leu cell^−1^).

### Respiration.

**FIG 4:**
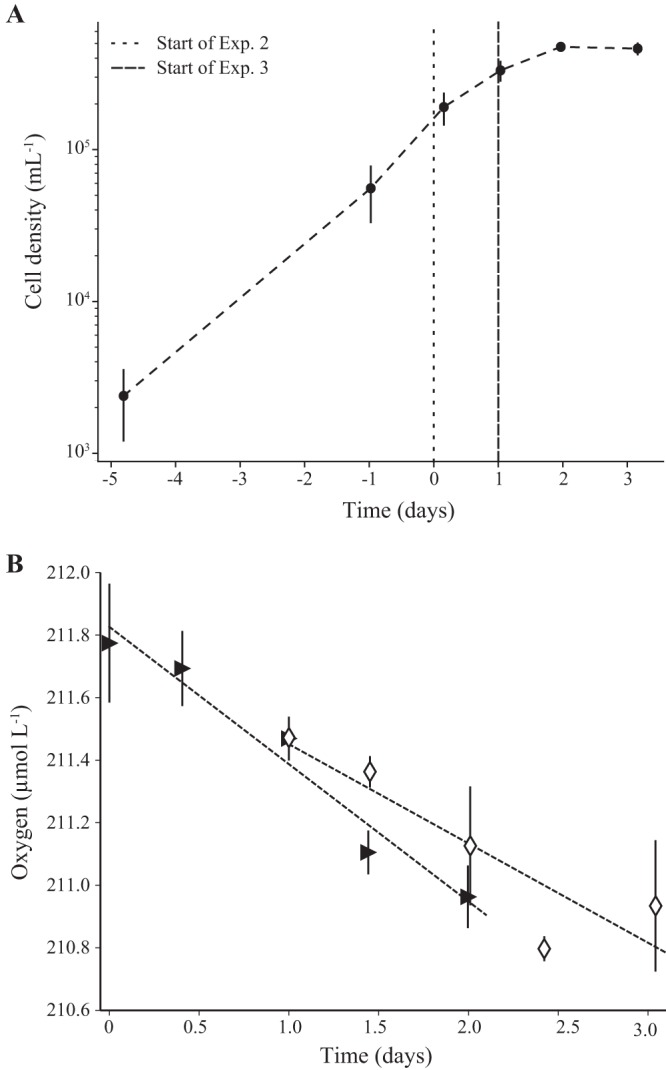
Respiration of strain HIMB114 during growth in batch culture. (A) Two oxygen respiration incubation experiments (experiments 2 and 3) started from a 10-liter batch culture of strain HIMB114 during late exponential phase of growth. (B) Triangles and diamonds represent the mean oxygen concentration for each time point in the second and third incubation experiment, respectively (Table 2, experiments 2 and 3). Error bars represent the standard deviations.

The rates of respiration were determined from three incubation experiments subsampled from the batch cultures (Table 2). Respiration was derived from linear regression fits to time course experiments in which the concentration of O_2_ was measured over 2-day incubation periods (Fig. 3A and 4). Although extensive measures were taken to thoroughly acid clean and rinse the glass bottles, HIMB114 cells were able to grow in the glass incubation bottles only for a period of about 2 days when growth rate and leucine incorporation declined, and the consumption of O_2_ was linear only over this initial ∼2-day period (Fig. 4). Respiration rates showed high reproducibility over the three incubation experiments (0.37 ± 0.06 μmol O_2_ liter^−1^ day^−1^ [mean ± SD; *n* = 3]) (Table 2) for cultures beginning with cell densities near 2 × 10^5^ ml^−1^ at the start of the incubations and increasing on average 2.5 times over the 2-day incubation period to about 5 × 10^5^ ml^−1^. Cell-normalized rates of respiration averaged ∼1 fmol O_2_ cell^−1^ day^−1^ when cultures were transitioning from exponential growth to stationary phase at a specific growth rate of approximately 0.5 day^−1^.

**TABLE 2:** Summary of experiments to measure respiration by the time-dependent consumption of dissolved O_2_ in late-log-phase batch cultures SAR11 strain HIMB114^*a*^

Expt	Growth rate (slope ± SE) (day^−1^)	Abundance (mean no. of cells [min, max]) (10^8^ cells liter^−1^)	Respiration		Production		Mean BGE [min, max] (%)
			μmol O_2_ liter^−1^ day^−1^ (mean [min, max])	fmol O_2_ cell^−1^ day^−1^ (mean ± 95% CI)	μg C liter^−1^ day^−1^ (mean [min, max])	fmol C cell^−1^ day^−1^ (mean ± 95% CI)	
1	0.15 ± 0.2	4.0 [3.5, 5.3]	0.36 [0.10, 0.61]	0.90 ± 0.65	0.41 [0.30, 0.52]	0.09 ± 0.03	9 [5, 26]
2	0.9 ± 0.2	3.3 [1.9, 4.8]	0.44 [0.27, 0.60]	1.32 ± 0.76	0.82 [0.70, 0.93]	0.27 ± 0.06	13 [10, 21]
3	0.5 ± 0.1	4.2 [3.3, 4.8]	0.32 [0.14, 0.50]	0.75 ± 0.44	0.77 [0.66, 0.87]	0.17 ± 0.04	17 [11, 32]

a Growth rates were calculated by linear least-squares regression of cell density over the 2-day incubation periods. Note that experiment 1 was conducted toward the transition out of log phase and into stationary-phase growth, with an associated flattening growth curve. Respiration was measured by linear least-squares regression of oxygen concentration with time over the 2-day incubations. Bacterial production values are 2-day means of daily, 2-h leucine incubations. Bacterial growth efficiency (BGE) was calculated as described in the text, assuming a respiratory quotient of 1 mol C:mol O_2_.

The rates of respiration were also derived based on changes in total organic C (TOC) within HIMB114 cultures over several days, measured at the start and end of the incubations of six replicate 10-liter batch cultures of strain HIMB114 (Fig. S1). The initial TOC concentration in the medium was 83 ± 2 μmol C liter^−1^ (mean ± SD; *n* = 6), while the final TOC concentration sampled 9 days later was 79 ± 3 μmol C liter^−1^ (mean ± SD; *n* = 6), resulting in a mean drawdown of TOC over the 9-day incubation of 4 ± 4 μmol C liter^−1^ (mean ± SD; *n* = 6). This is equivalent to approximately 5% of initial TOC. The resulting average rate of respiration was 0.44 ± 0.44 μmol C liter^−1^ day^−1^. This rate is very similar to the rate of respiration derived from O_2_ consumption (based on the 2-day incubation period), assuming a respiratory quotient of 1 mol C: mol O_2_ (0.37 ± 0.06 μmol C liter^−1^ day^−1^ [mean ± SD; *n* = 3]).

### Bacterial growth efficiency.

By combining the rates of bacterial production with the measured rates of respiration, we were able to estimate bacterial growth efficiency (BGE) for the HIMB114 batch cultures. BGE is defined as the ratio of the C production rate to the total C demand, which is the sum of bacterial production (BP) and bacterial respiration (BR): BGE = BP/(BP + BR). Combining these rate measurements resulted in a mean BGE of 13% with a 95% confidence interval of 10 to 21% estimated by a Monte Carlo simulation study (Table 2).

## DISCUSSION

Steady-state chemostat growth provides an ideal system to investigate the physiology and cellular properties of model microorganisms. The chemostat system allows the investigation of cellular physiology under controlled growth rate conditions, something unachievable using batch cultures. In this case, the steady-state growth achieved through the chemostat allowed us to simultaneously calculate the theoretical P demand as well as determine the actual PO_4_^3−^ uptake rate at a set rate of growth. Despite the inability to grow strain HIMB114 under P-limiting conditions, these experiments suggest that this isolate relies heavily on sources of P other than PO_4_^3−^ when grown on a natural seawater minimal medium. This finding is particularly intriguing considering that the HIMB114 strain has a complete high-affinity PO_4_^3−^ transport system (19), and so should have the full capacity to transport PO_4_^3−^ under dilute conditions. While inorganic PO_4_^3−^ is generally considered the preferred P source for marine bacteria (14), oligotrophic marine environments such as Kaneohe Bay in the tropical Pacific Ocean, where strain HIMB114 was isolated, typically have DOP concentrations an order of magnitude above inorganic phosphate concentrations (20). Thus, the ability to utilize components of the DOP pool to attain P may be competitively advantageous. In both batch and chemostat culture conditions, HIMB114 appeared to utilize an undetermined component of the DOP pool to meet its P growth demands, even when PO_4_^3−^ was amended to the media. Which component(s) of the DOP pool was utilized remains to be determined. However, one potential class of DOP compounds receiving recent attention are phosphonates, which are organic phosphonic acid derivatives containing a C-P bond and which make up 25% of the high-molecular-weight DOM pool (21). Evidence of the widespread distribution of genes for the transport and metabolism of phosphonates has been reported in marine microorganisms (22–24) including SAR11 (see Table S1 in the supplemental material) (17, 19), and there is precedent for the PO_4_^3−^-independent utilization of phosphonates in marine systems (25). In laboratory experiments with a defined growth medium, SAR11 subgroup Ia strain HTCC7211 was shown to utilize phosphonates as a source of P for growth (12). While the genome of strain HIMB114 encodes a complete phosphonate transport system similar to that of HTCC7211, it encodes a unique and sparse complement of genes for phosphonate metabolism (Table S1).

10.1128/mSystems.00218-18.2 TABLE S1 Presence of genes for phosphorus uptake and metabolism in selected publicly available SAR11 genomes sequenced from cultivated strains. Download Table S1, PDF file, 0.05 MB. Copyright © 2019 Grant et al. 2019 Grant et al. This content is distributed under the terms of the Creative Commons Attribution 4.0 International license.

At 14.2 amol P cell^−1^, the measured P cell quota for HIMB114 is close to that determined for SAR11 subgroup Ia strain HTCC7211 (10.9 amol P cell^−1^) grown on a P-limiting, defined medium (12), and fits well within the range of 10 to 22.6 amol P cell^−1^ measured for strains HTCC7211 and HTCC1062 grown on P-replete, defined medium (26). Using transmission electron microscopy coupled with X-ray microanalysis, Gundersen and colleagues found an empirical biovolume (*V*) power law relationship for cell P quotas of 126 *V*^0.937^ amol P cell^−1^, measured for 84 bacterial cells with a mean cell volume of 0.08 μm^3^ (range, 0.001 to 2.0 μm^3^) (27). Using this power law for HIMB114 cells suggests a cellular quota of 13 amol P cell^−1^, similar to the value measured in our study. At 1.237 Mbp and 2 P atoms per base pair, the small genome of strain HIMB114 yields a P content of 4.1 amol P cell^−1^. This calculation suggests that DNA alone accounts for approximately 29% of the cellular P quota, a finding roughly 2 to 3 times the 10 to 15% cellular P traditionally considered accounted for by DNA in a bacterial cell (28). Another significant pool for P is likely phospholipids, which have previously been measured to contain 2.5 amol P cell^−1^ for strain HIMB114 grown in PO_4_^3−^-replete seawater medium (29). Thus, nearly half of the P quota of the cell can be accounted for by only two macromolecular components: DNA and phospholipids. While not quantified in HIMB114 or other SAR11 cultures, RNA is typically the dominant molecular pool contributing to cellular P; typical total RNA-to-DNA mass ratios are infrequently below 2:1 (mass RNA:mass DNA), even for slowly growing bacteria (30), though more recent work has found a stable 1:1 RNA:DNA molar ratio for marine *Synechococcus* across a range of growth rates (31). Assuming a ratio of 1:1 RNA:DNA, an additional 4.1 amol P cell^−1^ can be accounted for by inclusion of cellular RNA pools. Hence, strain HIMB114 appears to have a similar cellular P concentration (0.16 M P) compared to other marine bacteria (0.1 to 0.2 M P) (32), and it would be difficult to further reduce this P demand unless these cells were able to substitute P-free lipids for phospholipids, as has been demonstrated for SAR11 subgroup Ia strain HTCC7211 (33). However, no genetic capacity for phospholipid substitution analogous to that found in the genome of strain HTCC7211 is apparent in the HIMB114 genome.

At 0.05 μm^3^, the peak of the distribution for cell volume of strain HIMB114 was consistent with the recently reported range of 0.015 to 0.058 μm^3^ for SAR11 subclade Ia isolates HTCC1062 and HTCC7211 (3). However, the mean value of the cell volume distribution (0.09 μm^3^) for HIMB114 was larger than anticipated, which can be at least partially attributed to elongated cells and chains of cells that increase in frequency as HIMB114 enters into stationary phase (Fig. 1). This phenomenon has been observed previously for SAR11 subgroup Ia strain HTCC1062 (5) and thus may be a broadly distributed, growth stage-dependent feature of SAR11 that has the potential to confound models and other measurements that rely on an average cell size or that are normalized per cell. For example, this phenomenon may contribute to the variability observed between bacterial production measured by leucine incorporation and cellular growth rate during the transition to stationary phase (Fig. 3).

The genome and membrane envelope are two essential components of a cell that cannot be continuously scaled down with cell size (6), and hence represent increasing fractions of total cell volume, or mass, for cell volumes below 0.05 μm^3^ (Fig. S5). This constrains the lower limit for a bacterial cell volume to about 0.004 μm^3^. We therefore propose a potential trade-off between nutrient acquisition strategies and P growth requirements for small cells. In oligotrophic, nutrient-limited environments, a high surface area-to-volume ratio should increase a cell’s ability to compete for dilute nutrients, giving small cells a distinct competitive advantage. However, there is an opposing force balancing this trend toward smaller cell size, namely, an increasing P requirement relative to cell mass necessary to maintain a given growth rate. This trade-off is reflected in the importance of P-sparing strategies employed by oligotrophic picocyanobacteria such as *Prochlorococcus* (34), and the noted prevalence and diversity of P acquisition- and metabolism-related genes found to be important across large ocean ecosystem regimes (15, 17). In addition to membrane lipid renovation (33), strategies employed by very small, diverse, and successful bacteria of the SAR11 clade to sustain cellular P demands and otherwise maintain sufficient net growth rates to numerically dominate surface marine waters will no doubt continue to provide interesting discoveries.

10.1128/mSystems.00218-18.7 FIG S5 Volume scaling of the fraction of total cell volume occupied by the genome (solid line) and the cell envelope (dashed line) for a theoretical bacterial cell. The cell size parameters were calculated assuming spherical cells, with the volume of the cell envelope calculated assuming a spherical shell with an envelope thickness of 20 nm. The genome volume was calculated assuming a genome length of 1.237 Mbp, and a DNA volume per base pair of 1 nm^3^. At the volume of the most frequent HIMB114 cell (0.05 μm^3^), the cell envelope and genome take up 26% and 2.5% of the cell volume, respectively. Download FIG S5, PDF file, 0.09 MB. Copyright © 2019 Grant et al. 2019 Grant et al. This content is distributed under the terms of the Creative Commons Attribution 4.0 International license.

The exceedingly high C-to-N stoichiometry of near 40 ± 14:1 (moles C:moles N) is well outside normally reported ranges for bulk marine particulate organic matter or C:N ratios of flow cytometrically sorted natural planktonic populations (maximum, 24.4; mean, 9.4 ± 3.6; *n* = 277) (35). Moreover, the resulting C:N:P cellular stoichiometry for HIMB114 would approach 300:7:1 (moles C:moles N:moles P), a finding inconsistent with previous estimates for members of the SAR11 clade. Such results are primarily driven by the exceedingly large C cell quota measured for strain HIMB114 in this study (50 ± 47 fg). The N cell quota for HIMB114 (1.4 ± 0.9 fg) is slightly lower than the minimum N cell quota of 1.6 fg N for natural bacteria reported by Fagerbakke et al. (32). Using the biovolume power law regression from Gundersen et al. (27) to derive cellular N quotas results in 2.4 fg N for a cell volume of 0.09 μm^3^ (mean volume measured in the current study); the same relationship yields a cellular C quota of ∼13 fg C. Tripp and colleagues previously estimated that SAR11 subgroup Ia strain HTCC1062 contained 5.8 fg C cell^−1^ for cells with a biovolume of 0.035 μm^−3^ (7), which scales to 14.9 fg C cell^−1^ for a cellular volume of 0.09 μm^3^. Similarly, Cermak and colleagues estimated that cellular C quotas varied between 12 to 16 fg for SAR11 strains HTCC1062 and HTCC7211 (36), which would scale to 24 to 48 fg C for strain HIMB114 when accounting for differences in cell volume. Zimmerman and colleagues measured the cellular C content of SAR11 strain HTCC1062 by filtering cells on the same nominal 0.3-μm-pore-size glass fiber filters as used in our study, yielding a cellular C quota of 32.2. fg C cell^−1^ (37). A recent study by White and colleagues combined a centrifugation method to concentrate cells with a dilution series regression approach to measure cellular elemental quotas in laboratory strains of SAR11 (26). Whereas their N and P cellular quota measurements closely matched those in the current study, they found that SAR11 subgroup Ia strains HTCC1062 and HTCC7211 contained ∼6.5 fg C cell^−1^ when grown under nutrient-replete conditions. Such comparisons suggest that the measurements of cellular C from the current study are likely overestimates. Although it remains unclear what factors may have contributed to these results, such findings may reflect the poor filtration retention efficiency of the filters utilized for these measurements. Regardless, accurate quantification of cellular C content of SAR11 cells remains imperative for future research efforts.

Although there are no published bacterial production measurements for any axenic SAR11 cultures, we can compare our values to measurements from planktonic marine ecosystems where SAR11 often dominate. The observed mean leucine incorporation rate from this study (4.2 × 10^−8 ^pmol Leu cell^−1^ h^−1^) is very close to that of the natural community mean dark leucine incorporation rates, normalized to nonpigmented cell counts, for station ALOHA in the North Pacific subtropical gyre of 5 × 10^−8 ^pmol Leu cell^−1^ h^−1^ (38), and falls at the lower end of the range measured for natural surface seawater communities along a transect off the Oregon coast (0.39 × 10^−7^ to 4.7 × 10^−7 ^pmol Leu cell^−1^ h^−1^) (39). Malmstrom and colleagues measured the contribution of naturally occurring SAR11 populations to bulk [^3^H]leucine incorporation rates using a combination of microautoradiography and fluorescence *in situ* hybridization (Micro-FISH) in the Northwest Atlantic Ocean (40). These authors reported that SAR11 accounted for a large fraction (50%) of the bulk leucine incorporation rates in surface waters, where they represented 25 to 35% (2 × 10 to 4 × 10^8^ cells liter^−1^) of the picoplankton population. The resulting SAR11-specific C production rates were estimated to be from 0.5 μg C liter^−1^ day^−1^ for an open-ocean Gulf Stream site, increasing to about 3 μg C liter^−1^ day^−1^ for a coastal location. del Giorgio and Cole compiled published marine ecosystem bacterial production measurements and reported global mean bacterial production rates of 2.41 ± 0.33 μg C liter^−1^ day^−1^ for the coastal ocean to 0.37 ± 0.054 μg C liter^−1^ day^−1^ for the open ocean (41). In the current study, the bulk C production rate measured for HIMB114 cultures was 0.6 ± 0.2 μg C liter^−1^ day^−1^, similar to those estimated by Malmstrom and colleagues (40) and typical of open-ocean, oligotrophic values reported by del Giorgio and Cole (41).

Leucine incorporation rates are used as a standard proxy for biomass production under the assumptions that protein is a major constituent of cell biomass and that leucine represents a relatively stable proportion of bacterial protein (42). This allows consistent comparisons of protein synthesis rates, and thus biomass production, across the wide spectrum of bacterial species capable of taking up leucine. It was somewhat surprising then to find that bacterial production measured by leucine incorporation varied little across a range of growth rates for strain HIMB114, with only a weak correlation between the two measures. While this could be due to the low precision (typically 15%) for both cell counts and leucine incorporation measures, it is also possible that protein production rates and cell division rates are uncoupled at short time scales under nonlinear batch growth.

We also measured O_2_-based rates of respiration, with rates averaging ∼0.4 μmol O_2_ liter^−1^ day^−1^. These low rates of respiration were highly reproducible, with a coefficient of variation of 16% between replicate incubations. Moreover, the measured O_2_-based respiration measurements agreed with the measured drawdown of organic carbon over the course of the incubations, which together with measured rates of bacterial production yielded estimates of BGE near 13%. Such results suggest that strain HIMB114 grows at efficiencies similar to those of other marine heterotrophic bacteria, despite features such as an exceptionally small, streamlined genome that might be expected to enable more efficient growth.

Steindler and colleagues have published the only other dissolved oxygen measurements from a SAR11 culture, wherein SAR11 subgroup Ia strain HTCC1062 cultures were measured using an oxygen optode (4). Although rates of respiration were not explicitly calculated in that study, over the initial 69-h period of incubation, the O_2_ concentrations declined by approximately 100 μmol O_2_ liter^−1^, equivalent to a rate of O_2_ consumption of ∼35 μmol O_2_ liter^−1^ day^−1^. Cell densities in the experiments of Steindler and colleagues were 3 orders of magnitude higher than the densities of our experiment; when normalized to cell density, the rate of respiration for strain HTCC1062 was approximately 0.35 fmol O_2_ cell^−1^ day^−1^, approximately one-third of the rate observed for strain HIMB114.

### Conclusions.

Although we were unable to create a state of P-limited growth or to determine what may be limiting strain HIMB114 when grown on a minimal seawater medium, we were able to rule out many of the common C, sulfur, and specific amino acid growth substrates that have been shown to enhance growth for other SAR11 cultures and permit their growth in a defined, artificial seawater growth medium (5, 7, 8, 11, 43). While this implies caution in extrapolating the results of culture-based studies from specific SAR11 isolates to the SAR11 lineage as a whole, it also suggests that exciting metabolic features that distinguish populations, ecotypes, and major SAR11 sublineages await characterization. One such feature, uncovered by using a continuous culture of HIMB114, is the apparent inability of this strain to fulfill its cellular P demand through the uptake of PO_4_^3−^ alone. Our findings support the idea that at least some members of the SAR11 clade rely on organic P to support growth, which is also supplemented by the use of cellular P-sparing adaptations such as lipid renovation (1). Despite potential methodological issues with the measurement of cellular C content, the N and P cell quotas, production, respiration, and cell size measurements reported here provide new information for scientists and modelers interested in understanding the impact of SAR11 cells on the ecology of the global ocean.

## MATERIALS AND METHODS

SAR11 strain HIMB114 was previously isolated from Kaneohe Bay on the northeastern shore of the island of Oahu in the tropical Pacific Ocean using a dilution-to-extinction approach (2, 44). It is a member of subclade IIIa that, based on genome comparisons, exhibits genus-level divergence from the comparatively well-studied members of subgroup Ia (i.e., “*Candidatus* Pelagibacter”) (19, 45, 46). Strain HIMB114 would not grow in the defined artificial seawater-based media previously published for SAR11 (5, 43), nor could we enhance its cellular yield by previous organic carbon, vitamin, and nutrient additions that have proven successful for other SAR11 strains (7, 8, 11) (data not shown). Thus, all experiments were performed in natural seawater-based minimal medium with seawater collected from the southern basin of Kaneohe Bay (21° 26.181′ N, 157° 46.642′ W). To make growth medium, surface seawater (200 liters) was filtered through prerinsed (10 liters sterile water, followed by 10 liters seawater) 0.1-μm-pore-size polyethersulfone (PES) membranes (AcroPak 1000; Pall Corp., Port Washington, NY, USA) into clean 10-liter polycarbonate (PC) carboys. Individual 10-liter batches of seawater were subsequently autoclaved for 2.5 h at 121°C and allowed to cool. For both batch and chemostat media (media termed “K-Bay”), the seawater base was amended with nitrate (3 μmol liter^−1^ NaNO_3_), NH_4_^+^ (3 μmol liter^−1^ NH_4_Cl), PO_4_^3−^ (0.1 μmol liter^−1^ KH_2_PO_4_), and a vitamin stock solution added at 10^−5^ dilution (10^−6^ dilution for the chemostat medium) (2). All chemicals were BioUltra grade (MilliporeSigma, St. Louis, MO, USA). The vitamin stock solution contained vitamin B_1_ (thiamine hydrochloride; 1 g liter^−1^), B_3_ (niacin; 0.1 g liter^−1^), B_5_ (pantothenic acid; 0.2 g liter^−1^), B_6_ (pyridoxine; 0.1 g liter^−1^), B_7_ (biotin; 1 mg liter^−1^), B_9_ (folic acid; 2 mg liter^−1^), B_12_ (cyanocobalamin; 1 mg liter^−1^), *myo*-inositol (1 g liter^−1^), and PABA (4-aminobenzoic acid; 0.1 g liter^−1^). After nutrient additions, small amounts of autoclaved-sterile Milli-Q deionized water were added to the K-Bay medium to replace water lost as a result of autoclaving, achieving a final salinity of 32. The medium was then sparged with CO_2_, followed by air, through three in-line Whatman (GE Healthcare Life Sciences, Chicago, IL, USA) vent filters (HEPA 0.3-μm glass fiber to 0.2-μm PTFE to 0.1-μm PTFE) to restore the inorganic C chemistry and to bring the medium pH to between 8.0 and 8.1, and stored at 4°C until use. The HIMB114 strain was grown in batch cultures at 26°C under low light (33 μmol quanta m^−2^ s^−1^) and a 12-h/12-h light/dark cycle in volumes ranging from 100 ml to 10 liters, as well as a 4-liter continuous culture chemostat system (described below).

### Chemostat.

**FIG 5:**
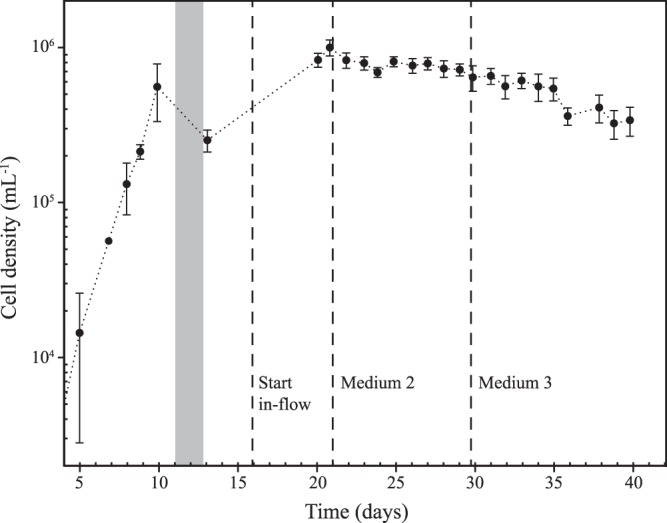
Chemostat continuous culture of SAR11 strain HIMB114. HIMB114 cells growing at 0.3 day^−1^ in chemostat continuous culture in natural seawater media at 26°C. The standard deviations of cell counts are indicated by error bars. The gray box indicates the time when filling the chemostat, while the dashed lines indicate when medium in-flow started and new 10-liter medium reservoirs were connected.

For continuous culture growth, a custom-built 4-liter PC chemostat was constructed using a narrow-mouth 4-liter PC bottle, four-port Teflon threaded cap, PC Luer connection fittings, and silicone tubing for the inflow of growth medium, culture overflow, air bubbling, and culture sampling ports (see Fig. S6 in the supplemental material). The chemostat was kept under positive pressure by bubbling with 0.1-μm-filtered air, which served to keep the culture well mixed as well as provide positive pressure for culture sampling. Medium was pumped from a 10-liter PC carboy continuously at 0.85 ml min^−1^ for a target dilution rate of 0.3 day^−1^. Overflow was continuously removed into a PC bottle used as an overflow container. To start the continuously growing culture, the chemostat was filled to 2 liters with the K-Bay chemostat medium (Table 3), inoculated with 5 ml of a growing HIMB114 culture, and allowed to grow in batch, where it reached an exponential growth rate of 0.75 day^−1^ for 10 days before medium in-flow was started (Fig. 5). After reaching the full 4-liter chemostat volume, cell densities stabilized at 7 × 10^5^ ml^−1^ after approximately 5 days, and the cells remained in continuous culture for 12 days or about 5 doubling times with continuous medium inflow and culture overflow (Fig. 5). The culture was grown in the chemostat for a total of 40 days; however, following the connection of the third 10-liter batch of new K-Bay chemostat medium at day 30, cell densities slowly declined to 3.5 × 10^5^ ml^−1^ by the end of the 40 days when the chemostat was turned off (Fig. 5).

**TABLE 3:** Nutrient concentrations for natural Kaneohe Bay seawater and the standard seawater-based medium used to grow strain HIMB114 in both batch and chemostat cultures^*a*^

Medium	Nutrient concn (μmol liter^−1^)					
	PO_4_^3−^	NO_3_^−^ + NO_2_^−^	NH_4_^+^	SiO_4_	DOC	TN
K-Bay seawater	0.05	<0.009	0.3	4.3	75	7.5
K-Bay standard medium	0.15	2.4	3.4	4.2	94	13.5

a Nutrient concentrations for dissolved phosphate (PO_4_^3−^), nitrate plus nitrite (NO_3_^−^ + NO_2_^−^), ammonium (NH_4_^+^), silicate (SiO_4_), total organic carbon (TOC), and total nitrogen (TN) for natural Kaneohe Bay (K-Bay) seawater with no additions and the standard seawater-based medium used to grow HIMB114 in both batch and chemostat cultures.

10.1128/mSystems.00218-18.8 FIG S6 Continuous culture of strain HIMB114. (Far left) Peristaltic pump; (center left) 4-liter narrow-mouth PC growth reservoir fitted with a four-port Teflon threaded cap; (center) 2-liter PC culture overflow container; (right) 10-liter PC carboy containing sterile growth medium. Download FIG S6, PDF file, 1.0 MB. Copyright © 2019 Grant et al. 2019 Grant et al. This content is distributed under the terms of the Creative Commons Attribution 4.0 International license.

### Cell enumeration and image analysis.

Culture cell counts were made by 4',6-diamidino-2-phenylindole (DAPI) staining and subsequent epifluorescence microscopy. Depending on culture density, culture samples between 2 to 10 ml were fixed with 20% electron microscopy-grade paraformaldehyde solution (Electron Microscopy Sciences, Hatfield, PA, USA) to a final fixative concentration of 0.4% and stored at 4°C overnight. DAPI was subsequently added to a final concentration of 5 μg liter^−1^ and incubated in the dark at room temperature for at least 20 min. Stained samples were filtered onto 25-mm-diameter, 0.2-μm-pore-size, black Nuclepore (GE Healthcare Life Sciences) or Isopore (MilliporeSigma) PC membranes, with a 0.8-μm-pore-size GN-4 (Pall Corp.) mixed cellulose ester backing filter. The filters were allowed to air dry for 15 min and either stored frozen (−20**°**C) or mounted in high-viscosity immersion oil on a glass slide for microscopic enumeration. At the volumes filtered, the precision of epifluorescence microscope cell counts was 10% to 20% for densities above 10^4^ ml^−1^. Cell number and size information (including cell lengths and widths) were calculated by image detection software from DAPI-stained epifluorescence images captured with a Retiga EXi FAST1394 camera (QImaging, Surrey, BC, Canada) at ×1,000 magnification.

Cell morphology was also visualized via scanning electron microscopy. HIMB114 cells grown in K-Bay medium to early stationary phase were fixed with glutaraldehyde (20%; Electron Microscopy Sciences), filtered onto 0.2-μm-pore-size PC membranes (Nuclepore), washed with sodium cacodylate buffer, postfixed in osmium tetraoxide, and subjected to sequential ethanol dehydration, critical point drying with CO_2_, and coating with gold/palladium. The preparations were viewed on a Hitachi S-4800 field emission scanning electron microscope with Oxford INCA X-Act EDS system.

### PO_4_^3−^ uptake.

The rates of PO_4_^3−^ uptake were measured in both batch and continuous culture using ^33^P radiotracer methods (18). Briefly, aliquots of growing culture (aliquots between 10 and 50 ml) were sampled from 50-ml PC tubes and spiked with ^33^P-orthophosphoric acid (158 Ci mg^−1^; PerkinElmer, Waltham, MA, USA) to a specific activity of 50 μCi liter^−1^. ^33^P-labeled cultures were incubated (typically for 4 to 8 h) under growth conditions identical to those of the parent cultures. Sample time points were collected by low-vacuum filtration of 5 ml onto 0.2-μm-pore-size, 25-mm-diameter PC Nuclepore membranes, each filter having been presaturated with unlabeled PO_4_^3−^ by the addition of 1 ml of high PO_4_^3−^ (0.1 mmol liter^−1^ PO_4_) seawater to each filter prior to sampling (see Text S1 in the supplemental material). Following filtration of ^33^P-labeled samples, filters were rinsed with 10 ml of 0.2-μm-filtered seawater. Along with the culture samples, 0.2-μm-filtered seawater controls amended with ^33^P radiotracer served as nonbiological blanks; these blanks were incubated and processed identically to samples. Phosphate uptake kinetics were also conducted for nine treatments of increasing PO_4_^3−^ concentration by the addition of 1 to 20 μl of concentrated unlabeled phosphate stocks (0.1 to 1 mmol liter^−1^ KH_2_PO_4_) to 11-ml chemostat culture samples. The treatments and filtered seawater controls were subsampled (2 ml) at five time points over 22 h of batch growth.

10.1128/mSystems.00218-18.1 TEXT S1 Supplemental methods. Download Text S1, PDF file, 0.6 MB. Copyright © 2019 Grant et al. 2019 Grant et al. This content is distributed under the terms of the Creative Commons Attribution 4.0 International license.

### Bacterial production.

Bacterial production was estimated based on the incorporation of tritiated leucine (^3^H-Leu) into protein using small-volume (1.5-ml) sample incubations based on the microcentrifugation method (47) (Text S1). Leucine incorporation rates were converted into C production rates using a standard 1.5 kg C mol Leu^−1^ conversion factor (39).

### Oxygen respiration.

Respiration rates were measured in batch cultures of strain HIMB114 based on time-dependent changes in the oxygen-to-argon (O_2_/Ar) ratios measured by membrane inlet mass spectrometry (MIMS) (48) (Text S1). Cultures growing in late exponential phase in 10-liter PC carboys were siphoned using silicone tubing into 70-ml clear glass serum bottles, allowed to overflow, capped with Teflon-lined rubber stoppers, and crimped-sealed. The glass bottles were extensively cleaned with Milli-Q DI water and 10% hydrochloric acid and finally autoclaved while filled with Milli-Q DI water before use. Sample bottles were filled in triplicate for each time point, with five time points sampled over a 2-day period, and in one case a 4-day period. Bottles were incubated under the same temperature and light conditions as the original cultures and either run immediately at each time point or the bacteria were killed by syringe addition of 100 μl of saturated mercuric chloride solution and analyzed at the end of the incubations.

### Monte Carlo simulation.

A Monte Carlo simulation study was conducted to quantify statistical errors in the oxygen-based respiration, bacterial production, and BGE measurements. Simulated data for O_2_ concentrations and leucine incorporation rates from the respiration and production experiments were generated by sampling (*n* = 10,000) from independent, normal distributions using sample means and variances based on experimental replicate measurements. Linear regression slopes were computed on the simulated O_2_ concentration samples with time to obtain simulated O_2_ respiration rates separately for each of three incubation experiments, with regression slopes bounded by zero (i.e., simulated data were prevented from indicating net O_2_ production with time). To convert from O_2_ and leucine units into carbon units, no uncertainty was assumed in the conversion factors, as we were attempting to estimate the statistical error from our measurement replication. BGE was calculated as indicated above, and 95% confidence intervals (2.5% and 97.5% quantiles) were calculated for each measurement by experiment (Table 2) and for the final reported mean BGE measure.

### Dissolved nutrients.

Samples for dissolved inorganic nutrients and TOC analyses were taken from both the original medium as well as the final spent medium at the end of culture incubations. Samples were collected in acid-washed, DI water-rinsed plastic (inorganic nutrients) or glass (TOC) containers, and stored frozen until analysis. Dissolved inorganic nutrient samples were analyzed on an Analytical Segmented Flow Injection AutoAnalyzer AA3 HR (SEAL Analytical Inc., Mequon, WI) for the determination of PO_4_^3−^, NH_4_^+^, nitrate plus nitrite (NO_3_^−^ + NO_2_^−^), silicate (SiO_4_), and total N. Samples for TOC were acidified and O_2_ purged to remove inorganic C and measured using high-temperature catalytic oxidation on a Shimadzu TOC-L (Shimadzu Scientific Instruments Inc., Columbia, MD).

### Cellular elemental analysis.

Six individual 10-liter cultures of strain HIMB114 were grown in batch for the purpose of collecting 20 liters of cultured cells onto triplicate Advantec GF-75, 25-mm-diameter glass fiber filters (Sterlitech, Kent, WA, USA), with a nominal pore size of 0.3 μm, for subsequent measurements of cellular C and N quotas. Filters were dried, pelleted, and analyzed using an elemental analyzer (CE440 elemental analyzer; Exeter Analytical, North Chelmsford, MA, USA). The batch cultures were filtered by slowly pumping the cultures from 10-liter carboys into large-volume filter towers containing combusted 25-mm GF-75 filters; the filtrate was retained in separate 10-liter collection carboys for subsequent microscopic analyses to assess the cellular retention efficiency of the filters. A total volume of 30 liters was filtered through each membrane filter: 20 liters from two separate 10-liter cultures, and 10 liters of culture filtrate containing cells that passed through the first filtration. The cell retention efficiencies of the filters declined in each successive 10-liter round from a mean retention of 37% in the first round down to 9% in the final third round of filtration from the filtrate. The overall cell retention rate for the full filtration procedure was 40%, resulting in an average of 5 × 10^9^ ± 2 × 10^9^ cells (mean ± SD; *n* = 3) on each filter. Preliminary tests indicated that procedural blanks were necessary to account for adsorption of noncellular dissolved C and N onto the filter (Text S1).

For the determination of cellular P, 4 to 5 liters of culture was collected by peristaltic pump filtration at a flow rate of 8 ml min^−1^ onto 0.2-μm-pore-size, 47-mm-diameter PC Nuclepore filters. All filtrations occurred in a walk-in cold room at 4°C for 8 to 10 h. Procedural blanks of spent medium were also made by filtering 50 ml of 0.2-μm medium filtrate, that is, the same medium in which the cultures were grown with cells removed, onto the 0.2-μm, 47-mm-diameter PC filters. Following filtration, filters were placed in acid-cleaned glass test tubes, covered with combusted aluminum foil, and stored at −20**°**C until analysis. Cellular P was quantified by a modification of the high-temperature combustion, colorimetric molybdate method (49) (Text S1).

10.1128/mSystems.00218-18.9FIG S7Batch growth and nutrient dynamics numerical model results (blue lines) for a batch culture of strain HIMB114, using phosphate as the sole source of phosphorus. (A) Phosphate (Pi) pool concentration; (B) rate of change of the Pi pool $\left(\frac{dP}{dt}\right)$ due to uptake by cells; (C) Pi pool turnover time (T_P_), with the gray horizontal solid line indicating the minimum turnover time of 50 days and the gray dashed horizontal line showing when the turnover time is 1 order of magnitude above the minimum (500 days); (D) culture biomass as phosphorus concentration, with actual P biomass calculated from cell counts from the culture (circles); (E) phosphate uptake rate dynamics, assuming phosphate as the sole source of phosphorus. The observed uptake rate value is plotted as a horizontal dashed gray line near the bottom of the *y* axis, measured late on day 7 at 0.004% total P day^−1^. This intersects the modeled rate on day 11.6, nearly 4 days after the observed rate. (F) Similar to panel C, but with Pi pool turnover time in units of years and on a linear scale. Colored areas indicate time windows for turnover times less than 1 (light blue), 10 (blue), and 50 (purple) years. For all panels, the gray dashed vertical lines indicate the times of highest uptake rate of phosphate, or equivalently the shortest turnover time. This corresponds closely to the actual timing of the measured Pi uptake experiments from the HIMB114 batch cultures, shown by the cell density circle closest to the vertical line in panel D. Download FIG S7, PDF file, 0.1 MB.Copyright © 2019 Grant et al.2019Grant et al.This content is distributed under the terms of the Creative Commons Attribution 4.0 International license.

10.1128/mSystems.00218-18.10 FIG S8 [^3^H]leucine incorporation by strain HIMB114 as a function of incubation time. Download FIG S8, PDF file, 0.09 MB. Copyright © 2019 Grant et al. 2019 Grant et al. This content is distributed under the terms of the Creative Commons Attribution 4.0 International license.
