# Supplementary figures and images for: Elemental Composition, Phosphorous Uptake, and Characteristics of Growth of a SAR11 Strain in Batch and Continuous Culture
Source: mSystems. 2019 May 21;4(4):e00218-18. doi: 10.1128/mSystems.00218-18 (PMC6589437; doi:10.1128/mSystems.00218-18)

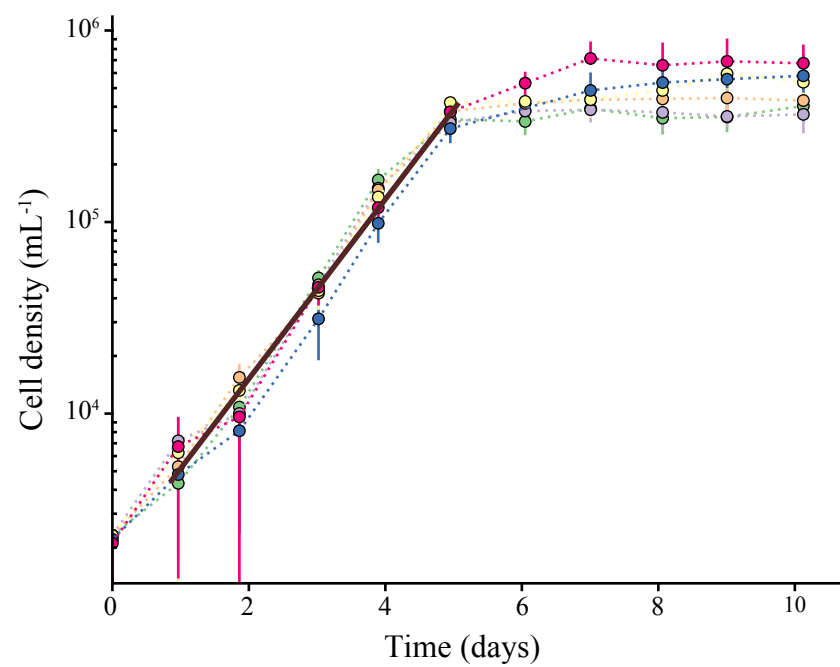

Supplement: FIG S1 [file mSystems.00218-18-sf001.pdf]

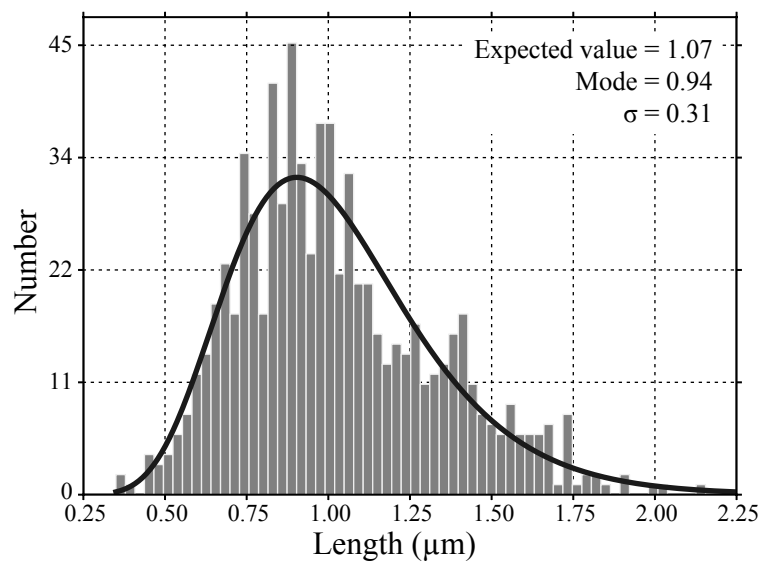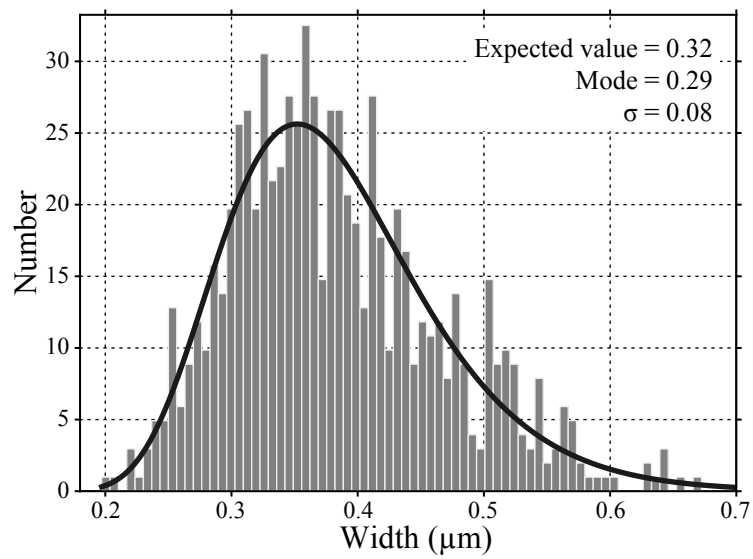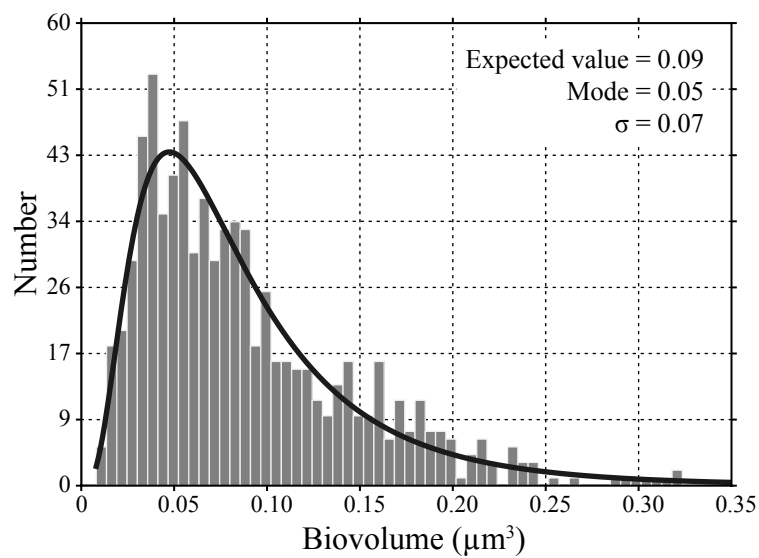

Supplement: FIG S2 [file mSystems.00218-18-sf002.pdf]

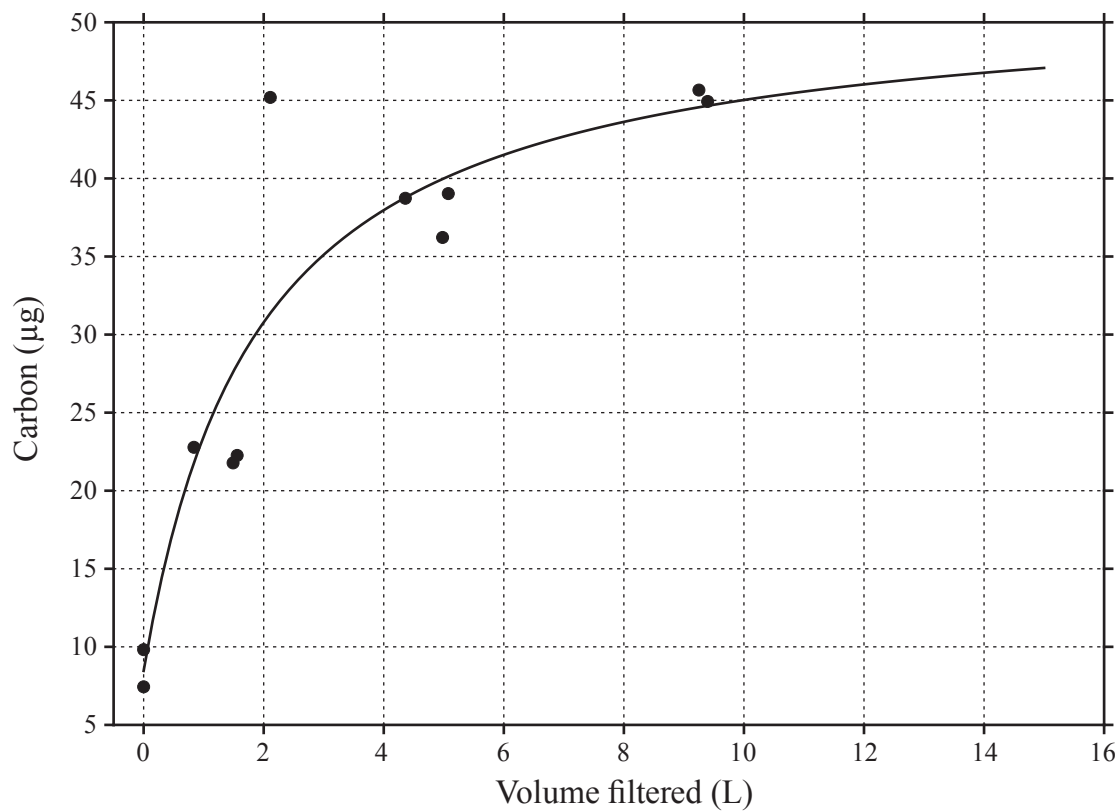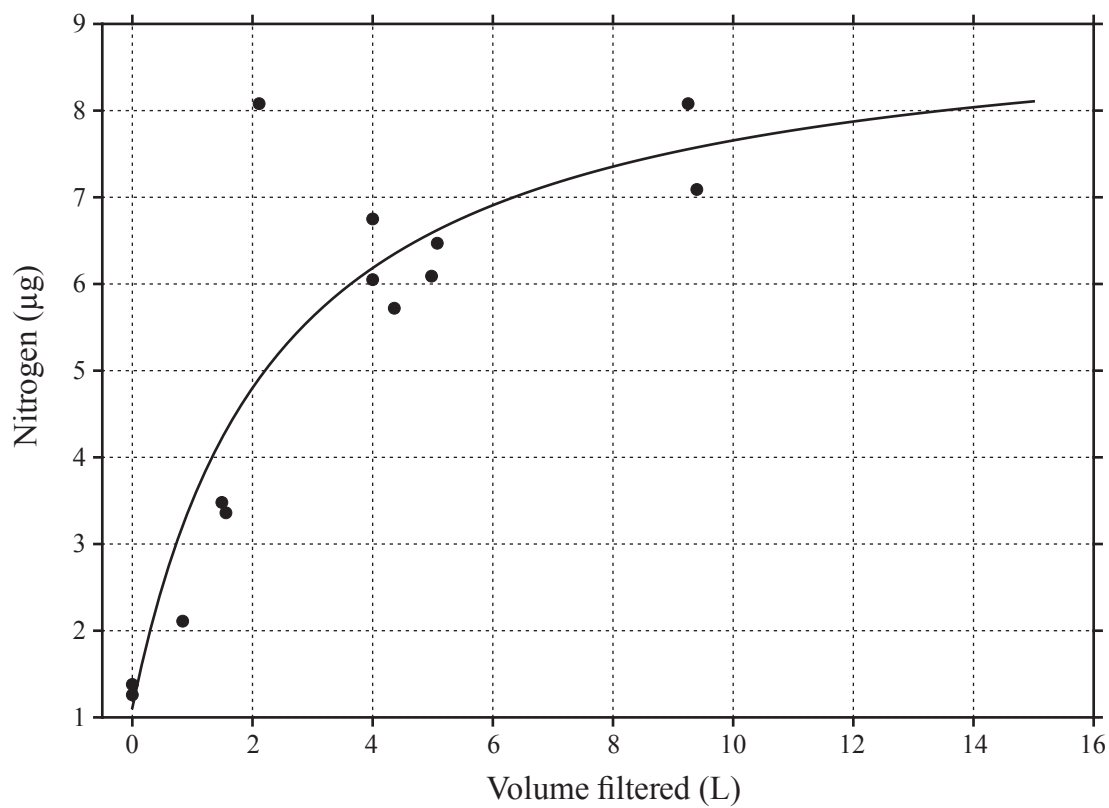

Supplement: FIG S3 [file mSystems.00218-18-sf003.pdf]

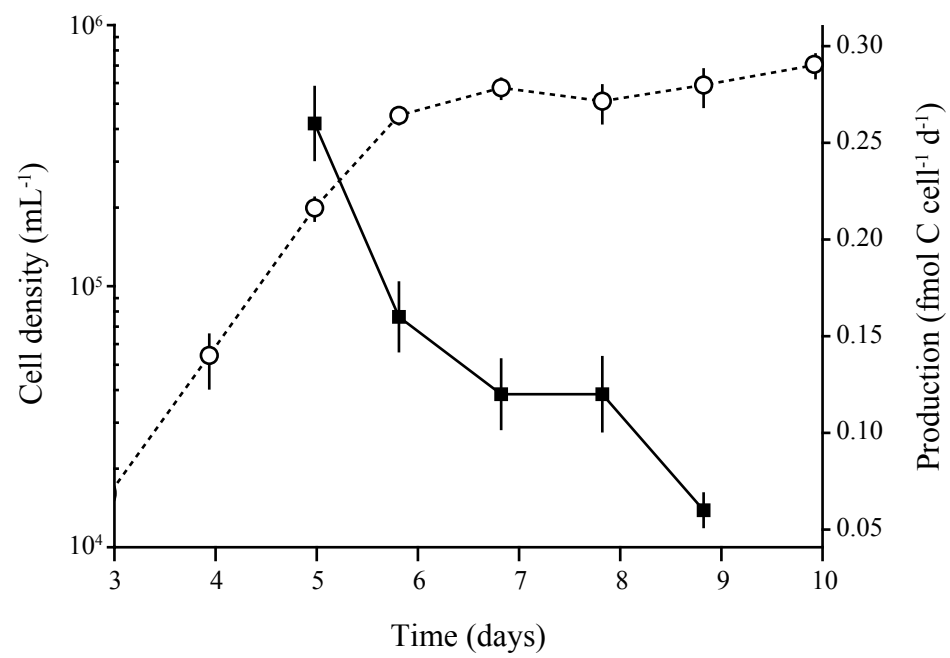

Supplement: FIG S4 [file mSystems.00218-18-sf004.pdf]

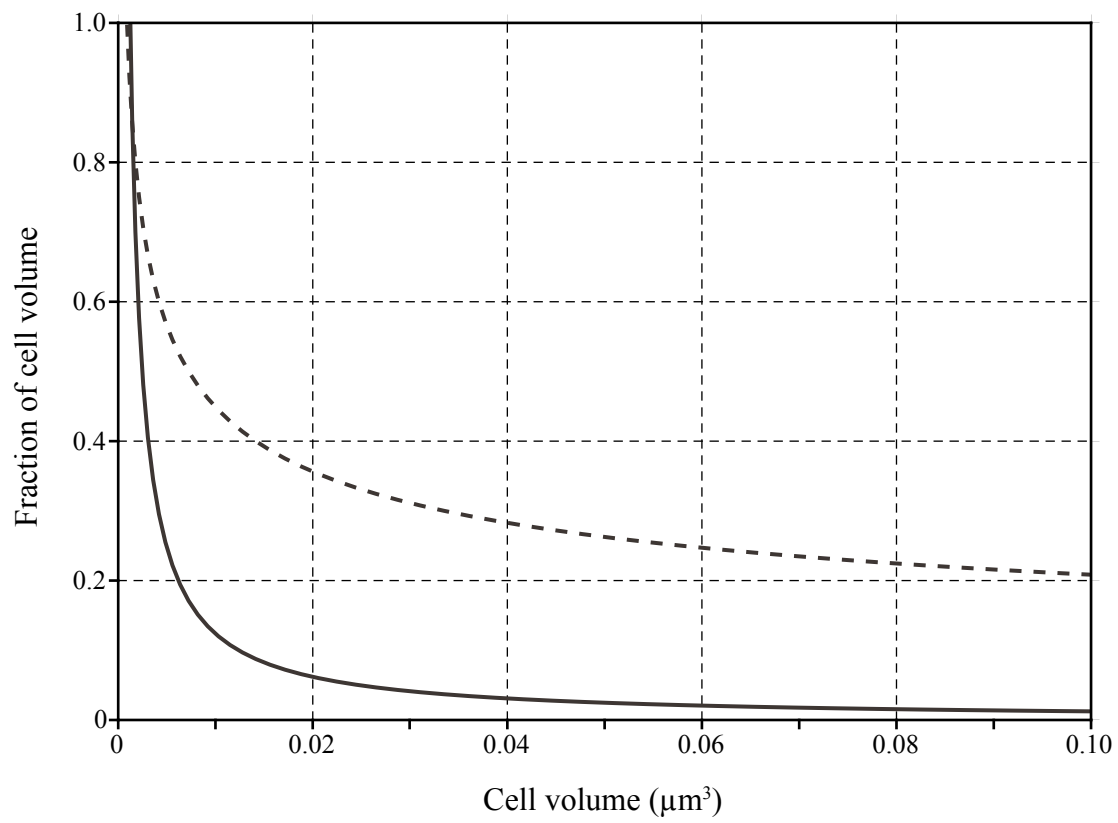

Supplement: FIG S5 [file mSystems.00218-18-sf005.pdf]

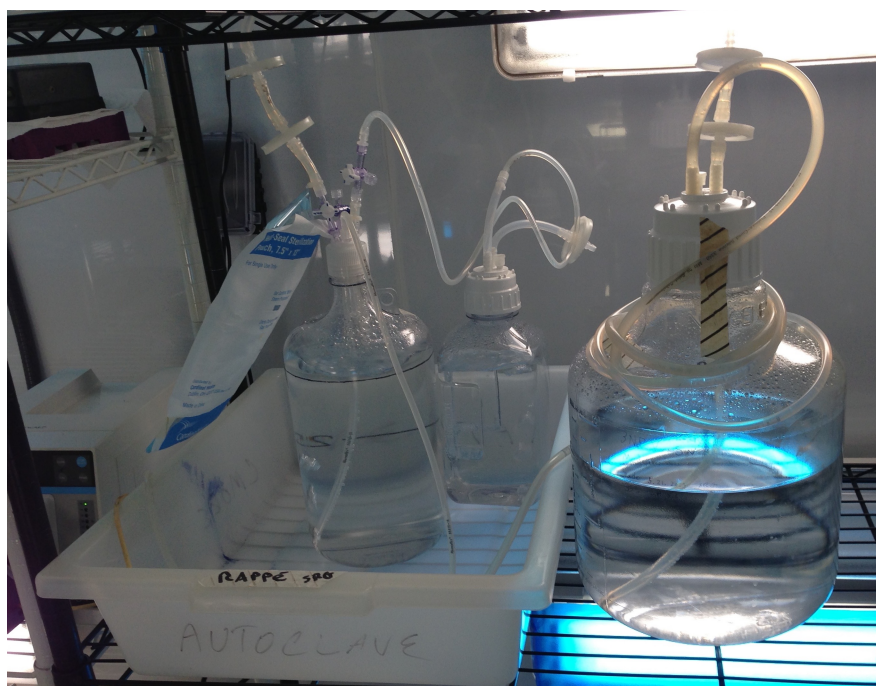

Supplement: FIG S6 [file mSystems.00218-18-sf006.pdf]

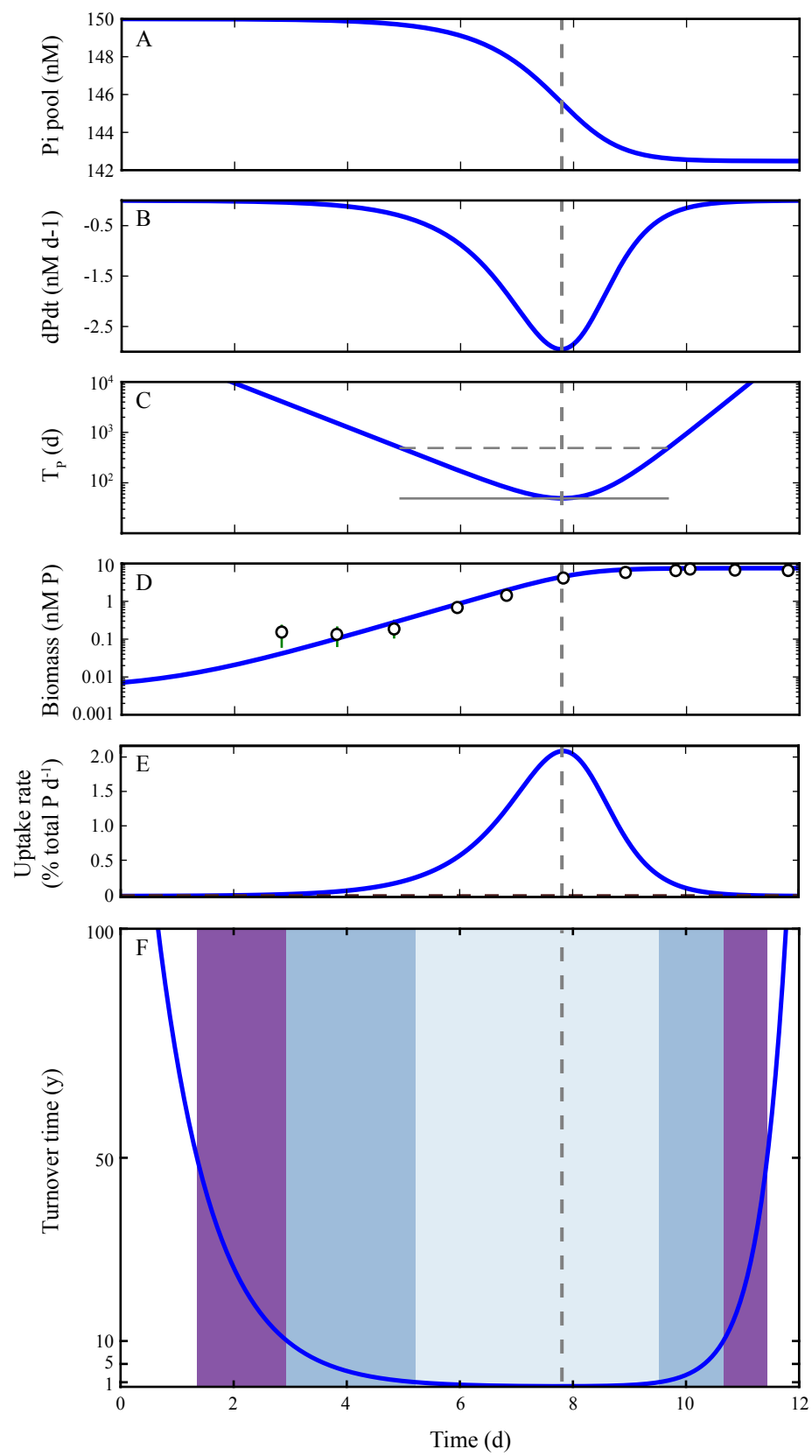

Supplement: FIG S7 [file mSystems.00218-18-sf007.pdf]

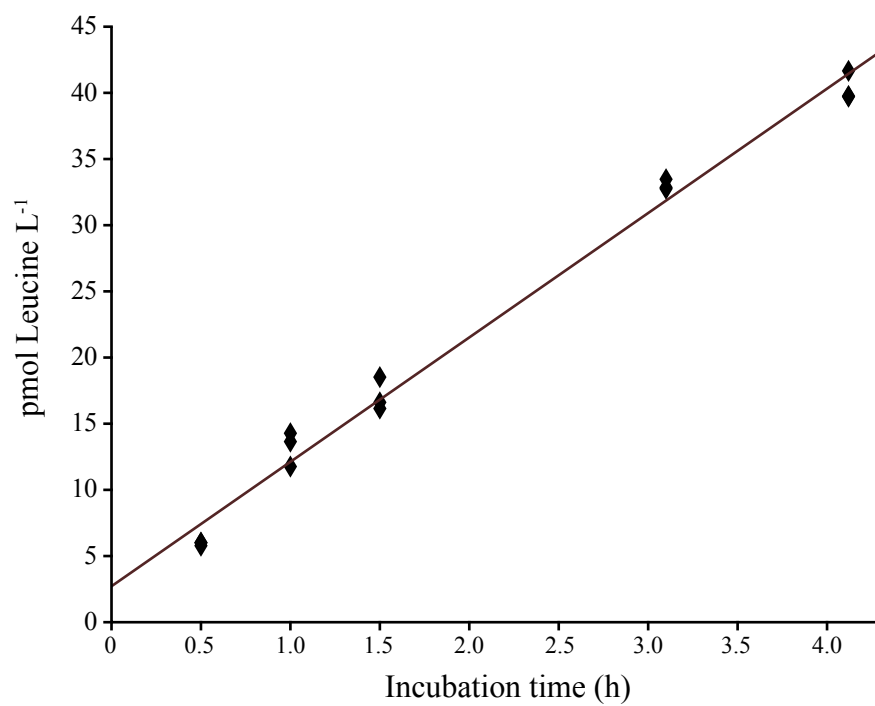

Supplement: FIG S8 [file mSystems.00218-18-sf008.pdf]
