# Supplementary material for: Elemental Composition, Phosphorous Uptake, and Characteristics of Growth of a SAR11 Strain in Batch and Continuous Culture
Source: mSystems. 2019 May 21;4(4):e00218-18. doi: 10.1128/mSystems.00218-18 (PMC6589437; doi:10.1128/mSystems.00218-18)
Supplement: TABLE S1 [file mSystems.00218-18-st001.pdf]

Table S1. Presence of genes for phosphorus uptake and metabolism in selected publicly available SAR11 genomes sequenced from cultivated strains.

Strain	Lineage	Source	pstS phosphate transport	phoBU phosphate regulation	phnCDE phosphonate transport	phnAGHI JKLMNX phosphonate metabolism	ppx/ppk polyphosphate metabolism
HIMB114	IIIa	Subtropical North Pacific	+	+	+	phnAX	-
HIMB58	IIb	Subtropical North Pacific	+	+	-	-	-
HIMB59	V	Subtropical North Pacific	+	+	+	phnAX	-
HIMB5	Ia	Subtropical North Pacific	+	+	-	-	-
HTCC7211	Ia	Sargasso Sea, Atlantic	+	+	+	+	+
HTCC9565	Ia	Temperate North Pacific	-	phoH	-	-	-
HTCC1002	Ia	Temperate Coastal Pacific	-	phoH	-	-	-
HTCC1062	Ia	Temperate Coastal Pacific	+	+	-	-	-
