# Supplementary material for: Elemental Composition, Phosphorous Uptake, and Characteristics of Growth of a SAR11 Strain in Batch and Continuous Culture
Source: mSystems. 2019 May 21;4(4):e00218-18. doi: 10.1128/mSystems.00218-18 (PMC6589437; doi:10.1128/mSystems.00218-18)
Supplement: TEXT S1 [file mSystems.00218-18-s0001.pdf]

Supplemental Methods

Cell enumeration and image analysis

Cell biovolume (V) was calculated from cell length (L) and width (W) using a bulk geometric function for a prolate spheroid, $V = \frac{\pi}{6}W^2L$. Although the bulk geometric function for a prolate spheroid has been shown to overestimate volumes for objects with similar shape morphology to HIMB114 by about 50%, it is likely the most appropriate bulk formula to use, and is better than using an equation for a cylinder with hemispherical caps, which can overestimate volumes for these shapes by 100% (Sieracki, Viles, & Webb, 1989).

Phosphate uptake

Control tests were made for the non-biological adsorption of ^{33}P-tracer to both Nuclepore PC and Supor PES membranes (Pall Corp.) with and without the high-phosphate pre-saturation step. PC membranes were found to be superior to PES membranes for lowering control background, retaining just 0.0001% and 0.05% of the total ^{33}P activity with and without the high-phosphate pre-saturation step, respectively. The Supor PES membranes retained significantly more of the ^{33}P-phosphate radiotracer, 0.07% and 0.34% of the total activity with and without the high-phosphate step, and with much higher variance between replicates than for the PC membranes. Therefore, PC membranes were chosen as the preferred filters for reducing both the background and sample variance for ^{33}P-phosphate uptake measurements.

Batch growth and nutrient dynamics model

A numerical model to describe the dynamics of batch culture growth was constructed to provide insights and theoretical comparisons for the measured PO_4^{3-} uptake rates and turnover

times under batch growth conditions. The model is cast in units of P concentration, in order to describe the uptake of PO_4^{3-} and growth of the culture with the assumption that PO_4^{3-} is the sole source of P for the culture.

The prognostic equation for the culture cell density (x) is described by a second order logistic equation:

$$\frac{dx}{dt} = \mu \cdot v \cdot x \cdot \left[1 - \left(\frac{x}{x_{max}}\right)^n\right]$$

where n is the exponential constant for logistic growth, which we set to 2 after investigating the growth curves for n = 1 to 4 (an exponent higher than 1 is needed to correctly model the observed, rapid transition from exponential to stationary phase); x_{max} is the observed maximum cell density for the culture; v is the rectangular hyperbolic Monod function for phosphate limited growth, which never came into effect here because the phosphate pool never approached, within an order of magnitude, the assumed phosphate half-saturation constant for growth of $K\mu$ = 1 nM P. The growth rate function, μ, is used to describe the transition from the lag phase to the exponential maximum growth rate phase, and is also described by a logistic equation:

$$\frac{d\mu}{dt} = \frac{1}{\tau} \cdot \mu \left[1 - \left(\frac{\mu}{\mu_{max}}\right)\right]$$

where τ is the timescale for the lag phase transition to exponential growth (1 d), and μ_{max} is the observed exponential phase growth rate. To convert from cell density to P-based biomass units, the measured P cell quota (Q_P) is assumed to be constant throughout the growth curve, and the

growth of cell biomass is directly coupled to the uptake of P from the phosphate pool:

$$\frac{dp}{dt} = -\frac{dx}{dt} \cdot Q_P$$

with observed initial concentrations of P and x used to initialize the model. The model was stepped forward with a time step of 30 minutes for 12 days using a simple Newton numerical scheme.

Because of the inherent non-linear dynamics of batch growth conditions, the theoretical nutrient uptake rates and turnover time may be quite dynamic, potentially changing by an order of magnitude on a daily timescale, making the interpretation of a measured rate on any particular day difficult. Results from the model matched reasonably well with the observed growth curve, and also confirmed that the time of maximum PO_4^{3-} uptake rates (Fig. S7E) and minimum turnover time (Fig. S7C) occur at the end of the exponential phase of growth (Fig. S7D). This corresponded quite closely to when the PO_4^{3-} uptake measurements were measured on this culture (Fig. S7D). The maximum theoretical specific uptake rate calculated by the model was 2×10^{-2} d^{-1}, or 3 nmol P L^{-1} d^{-1}, compared to the observed specific rate of 4×10^{-5} d^{-1}, or 6 pM P L^{-1} d^{-1}, measured very close to this time. The theoretical uptake rate falls off exponentially on either side of the maximum (Fig. S7E). The model also indicates that the minimum turnover time for the PO_4^{3-} pool, occurring on day 7.8, should be close to 50 days, again increasing exponentially around the minimum. The actual turnover time measured at the end of day 7 was 70 years (range from 50 to 150), about 500 times the minimum turnover time. Even if the lower estimate of 50 years is used, the timing would need to be off by over 3.5 days to measure a turnover time of that scale (Fig. S7).

Knowing what range of PO_4^{3-} turnover times to expect on a theoretical basis is quite difficult in batch cultures because of the non-linear dynamics, for which there is no clearly superior choice of functional parameterization for modeling batch culture growth (Zwietering et al., 1990). This creates a large uncertainty, of likely an order of magnitude, in the modeled uptake rates, which are particularly sensitive to the timing within the growth curve. Nevertheless, the turnover time was measured at what should be quite close to the time in the growth curve that would coincide with the minimum turnover time for PO_4^{3-}, and yet the measured turnover times were at least 500 times larger than expected.

Bacterial production

Typically, quadruplicate 1.5 mL sample volumes and duplicate blanks were added into 2 mL microcentrifuge tubes, followed by 2 μL of ^{3}H-3,-4,-5-Leucine (106 Ci mmol^{-1}; 5 mCi mL^{-1}; PerkinElmer) to a final concentration of 60 nmol Leu L^{-1}, mixed well, and incubated for 2.5 h under the same temperature and light conditions as the original cultures. Blanks were killed with trichloroacetic acid (TCA, 5% final) before the addition of ^{3}H-Leu. Incubations were stopped by the addition of TCA (5% final, 4 °C), centrifuged (14,000 rpm at 4 °C for 15 min), rinsed with 1 mL 5% TCA (4 °C), centrifuged again (14,000 rpm at 4 °C for 5 min), then rinsed with 1 mL 80% ethanol (4 °C) and centrifuged (14,000 rpm at 4 °C for 5 min). Pellets were allowed to dry overnight at room temperature in a fume hood before adding 1 mL of scintillation cocktail (UltimaGold LLT; PerkinElmer), vortexed, and allowed to sit for at least four days before making final activity counts (PerkinElmer Tri-Carb Liquid Scintillation counter), as the activity was observed to increase over the first two days after adding cocktail. Activity counts were converted to leucine concentration based on the specific activity of the isotope and

calibrated to a ^3H standard. Prior to using single 2.5-hr time point incubations, the linearity of ^3H-Leu incorporation by the HIMB114 culture was tested over 4 h and found to be quite linear over that time period (Fig. S8).

Oxygen respiration

Oxygen concentration measurements were made based on the mass spectrometric determination of the ratio of oxygen to argon. Briefly, the seawater sample was continuously pumped across a permeable membrane under vacuum, allowing dissolved gasses to diffuse across the membrane that were detected by an in-line mass spectrometer. An equilibrated seawater standard was used for calibration, and the oxygen concentration was calculated by the change in the O_2/Ar ratio referenced to the initial, time zero, oxygen concentration. Oxygen respiration rates were then calculated by linear least squares regression of oxygen concentration versus incubation time over two-day periods.

Cellular Elemental Analysis

Carbon and Nitrogen. Preliminary tests of filtration methods indicated that filtration by even the lowest of vacuum pressure retained undetectable cells, while filtration by gravity retained at most 50% of cells. This was slightly better than very slow (5 mL min^{-1}) peristaltic pump filtration, which was comparable to the rate of filtration by gravity. Because of the very slow filtration rates by gravity in combination with the large volumes that needed to be filtered, filtrations were conducted in a 4 °C walk-in cold room in order to stop cellular metabolism, and carried out over a period of 8 days of continuous gravity filtration. Pump speeds were adjusted to keep pace with the gravity filtration rates, which started at 3.5 mL min^{-1} and gradually slowed to

2 mL min^{-1} by day 5. Once filtration rates slowed to below 1 mL min^{-1} (day 8) the filtration was stopped.

For procedural blanks, batch cultures were 0.2 µm-filtered twice to remove all cells (0.2 µm pore-size, 47 mm-diameter Nuclepore PC membrane followed by 0.22 µm pore-size, Sterivex-GP PES membrane) and the filtrate used to construct dissolved carbon and nitrogen blank saturation curves by filtering 0, 1, 2, 5, and 10 L of sterile-filtered media through combusted GF-75 filters. These filters were then analyzed for carbon and nitrogen along with the sample filters (Fig. S3).

Phosphorus. Tubes containing sample filters were combusted for 9 h at 450 °C in order to convert organically bound phosphorus to inorganic phosphate, which was then extracted in 10 mL of 0.15 M HCl for 1 h at room temperature. Sub-samples (5 mL) of the acid extract were reacted with 0.5 mL of a molybdate mixed reagent solution to develop the blue phospho-molybdate complex. The molybdate mixed reagent was made by combining 10 mL of 30 g L^{-1} ammonium paramolybdate solution, 25 mL of 5N sulfuric acid, 10 mL of 5.4 wt.% ascorbic acid solution, and 5 mL of 1.7 g L^{-1} potassium antimony-tartrate solution. After allowing one h for color development, absorbance was measured on a spectrophotometer at 880 nm using a 1 cm path length quartz cell. Phosphorus concentrations were calculated using a phosphate standard curve, with the mean absorbance of the media procedural blanks subtracted from the sample absorbance.

Supplemental References

Sieracki ME, Viles CL, and Webb KL. 1989. Algorithm to estimate cell biovolume using image analyzed microscopy. Cytometry 10:551–557.

Zwietering MH, Jongenburger I, Rombouts FM, Vantriet K. 1990. Modeling of the bacterial-growth curve. Appl Environ Microbiol 56:1875–1881.
